# Performance of IMPACT, CRASH and Nijmegen models in predicting six month outcome of patients with severe or moderate TBI: an external validation study

**DOI:** 10.1186/s13049-014-0068-9

**Published:** 2014-11-19

**Authors:** Marek Majdan, Hester F Lingsma, Daan Nieboer, Walter Mauritz, Martin Rusnak, Ewout W Steyerberg

**Affiliations:** Department of Public Health, Trnava University, Faculty of Health Sciences and Social Work, Univerzitne namestie 1, Trnava, 91701 Slovakia; International Neurotrauma Research organization (INRO), Moelkergasse 4/3, Vienna, A-1080 Austria; Department of Public Health, Erasmus MC, Dr. Molewaterplein 50, Rotterdam, 3015 CE the Netherlands

**Keywords:** Prognostic model, External validation, Traumatic brain injury, Outcome prediction

## Abstract

**Background:**

External validation on different TBI populations is important in order to assess the generalizability of prognostic models to different settings. We aimed to externally validate recently developed models for prediction of six month unfavourable outcome and six month mortality.

**Methods:**

The International Neurotrauma Research Organization – Prehospital dataset (INRO-PH) was collected within an observational study between 2009-2012 in Austria and includes 778 patients with TBI of GCS < = 12. Three sets of prognostic models were externally validated: the IMPACT core and extended models, CRASH basic models and the Nijmegen models developed by Jacobs et al – all for prediction of six month unfavourable outcome and six month mortality. The external validity of the models was assessed by discrimination (Area Under the receiver operating characteristic Curve, AUC) and calibration (calibration statistics and plots).

**Results:**

Median age in the validation cohort was 50 years and 44% had an admission GSC motor score of 1-3. Six-month mortality was 27%. Mortality could better be predicted (AUCs around 0.85) than unfavourable outcome (AUCs around 0.80). Calibration plots showed that the observed outcomes were systematically better than was predicted for all models considered. The best performance was noted for the original Nijmegen model, but refitting led to similar performance for the IMPACT Extended, CRASH Basic, and Nijmegen models.

**Conclusions:**

In conclusion, all the prognostic models we validated in this study possess good discriminative ability for prediction of six month outcome in patients with moderate or severe TBI but outcomes were systemically better than predicted. After adjustment for this under prediction in locally adapted models, these may well be used for recent TBI patients.

## Introduction

Traumatic brain injury (TBI) is the leading cause of disability and mortality among young individuals in high-income countries and it is a rising public health issue in low-income countries [1]. Partly because of the heterogeneity of the disease in terms of cause, pathology and severity considerable uncertainty may exist in the expected outcome of patients [2]. Many studies reported on univariate associations between predictors and outcome after TBI and further research has been devoted to combine single predictors into prognostic models [1]. Such models have a relatively wide applicability ranging from clinical settings and research to providing information on expectations to relatives of patients [1-3]. Facilitating better severity classification, stratification in the context on Randomized Clinical Trials (RCTs) and setting baseline for clinical audits could be seen as the prime roles of prognostic models in TBI [2].

There is a relatively large number of prognostic models for outcome of TBI which utilize a variety of predictors and outcome measures. A systematic review published in 2006 identified 53 reports, which included a total of 102 models [4], but their quality was mostly poor [2,4].

Lack of external validation represents one of the main shortcomings of most models. External validation on different TBI populations, preferably collected in a multicentre setting is important in order to assess the generalizability of prognostic models to different settings [2,4]. It is reasonable to expect that even though biologically the prognostic factors should be the same for all patients, the association could differ depending on medical care received, which may differ by place and evolve over time [4]. Specifically, predictions based on data from decades ago may overestimate the incidence of poor outcomes for current clinical settings.

The International Mission for Prognosis and Analysis of Clinical Trials in TBI (IMPACT) [5] and the Corticosteroid Randomization After Significant Head Injury (CRASH) studies [6] are the most established prognostic models currently. Both were developed on large patient samples from multiple countries with state of the art methodology and have been externally validated beyond the process of model development [7-10]. More recently, a set of prognostic models was developed by Jacobs et al [11] using patient data from Nijmegen, the Netherlands. External validation in a fully new setting has not yet been performed (besides the external validation during development). Further validation of these 3 sets of models in recent patients is important to assess generalizability, since differences in treatment and health care organization in different populations have been suggested [10].

The aim of this study was to externally validate the IMPACT, CRASH and Nijmegen models for the prediction of six month unfavourable outcome and six month mortality in a recent observational patient cohort from Austria in 2009-2012.

## Methods

### Patients and population

The International Neurotrauma Research Organization (INRO) based in Vienna aims to improve the care and outcome of patients with TBI primarily in Austria, the wider region of Central Europe, and in countries of the South-Eastern European region. INRO led a prospective observational study focusing on prehospital and early hospital care of patients with TBI in Austria between 2009 and 2012. The study has been approved by the Ethical Committees of all participating centres and have been performed in accordance with the ethical standards laid down in the Declaration of Helsinki and its later amendments. All patients with Glasgow Coma Scale score (GCS) < = 12 within 48 hours after the accident and/or Abbreviated injury scale (AIS) [12] score of head >2 were included in the study. Data on demographic characteristics, injury type and severity, prehospital treatment, trauma room treatment, surgical procedures, CT scans, Intensive Care Unit (ICU) based treatment (first 5 days) and outcome (at ICU discharge, hospital discharge and six month after injury) were recorded. The Extended Glasgow Outcome Scale (GOSE) was used to categorize the outcome at hospital discharge (assessed by a local study investigator) and at six months after injury (assessed by telephone interview by a trained physician). The follow-up rate six months after injury was 70%. We analysed data from 778 patients from 16 centres.

### Prognostic models

For this study three sets of prognostic models (eight models in total) were selected. These models fulfilled requirements for prognostic models in TBI with respect to the study population, predictors, outcome, model development and validation [2,3]. All models were designed to predict six month mortality and six month unfavourable outcome in patients with TBI. Unfavourable outcome was defined as GOS of 1-3 (death, vegetative state or severe disability) in all models. Table 1 presents a comparative description of models and their development characteristics.

**Table 1:** **Description of prognostic models used in the validation study and their development characteristics**

**Model**	**N at development**	**Development setting**	**Data collection period**	**Predictors used in model**	**Outcome measures**
**IMPACT Core**	8,509	8 RCTs, 3 observational studies, multi-centre, multi-country	1984-1997	Age, GCS motor score, pupillary reactivity	6 month mortality, 6 month unfavourable outcome
**IMPACT extended**				Core model predictors and CT classification, hypoxia, hypotension, tSAH, EDH	
**CRASH basic**	10,008	RCT, multi-centre, multi-country	1999-2004	Age, GCS, pupillary reactivity, presence of major extra-cranial injury	14 day mortality, 6 month unfavourable outcome
**Nijmegen Clinical and demographic**	700	Observational study, single centre (Radboud University, Nijmegen, the Netherlands)	1998-2006	Age, pupil s reactivity, GCS, hypotension	6 month mortality, 6 month unfavourable outcome

RCT = Randomized Clinical Trial; GCS = Glasgow Coma Scale; CT = Computed Tomography; tSAH = Traumatic Subarachnoid Haemorrhage; EDH = Epidural hematoma.

Within the IMPACT study [13] three models of increasing complexity were developed (core model, extended model and lab model which added glucose and haemoglobin levels to predictors in the extended model). We focused on the core and extended models because the lab parameters were not available in the INRO-PH dataset.

The CRASH prognostic model [6] has two variants. One is for patients in low and middle income countries and the second for patients in high income countries. For the purposes of our study the models for high income countries were used. Only the basic model was validated since the CT predictors as they were defined in the CRASH CT models were not available in the INRO-PH dataset. Major extra-cranial injury in the INRO-PH dataset was defined as being serious or worse (2 or more scoring points) according to the AIS.

The Nijmegen models [11] were developed using the Radboud University Brain Injury Cohort Study (RUBICS) dataset, which enrolled patients between 1999-2006 [14]. Two prognostic models were developed. In our study only the demographic and clinical models were validated because the predictors as defined in the CT models were not available in our validation dataset.

The IMPACT models were developed using both RCT and observational data from a total of 11 studies, the CRASH and Nijmegen models were developed using data from a single study – an RCT and an observational study, respectively. The IMPACT and CRASH datasets were considerably larger than the dataset used to develop the Nijmegen models. Age, GCS (either summary or motor score only) and pupillary reactivity as predictors were common for all validated models. The Nijmegen models used the most recent data for development.

### Statistical analysis

To investigate the difference in predictor effects between the developed models and the validation population we fitted logistic regression models containing the predictors of the models in the validation population (using second level of customization). The estimated ORs of the different predictors were compared to the ORs of the original models. The external validity of the models was assessed using analysis of discrimination and calibration, following previous studies [3,7,10,13].

To assess the discrimination (ability to distinguish between survival and death or favourable and unfavourable outcome) the area under the receiver operating characteristic curve (AUC) was used. For a model with perfect discrimination the AUC = 1.0. An AUC of 0.5 indicates that the discriminative ability of the model is no better than by chance. For all models AUCs at external validation (AUC_VAL_) were calculated along with AUCs of the models refitted on the INRO-PH dataset (AUC_REFIT_). Confidence intervals are presented for each AUC_VAL_ calculated non-parametrically, using the bootstrap method of the ‘ci.auc’ function within the R package pROC [15].

Calibration evaluates the agreement between observed and predicted outcomes and was assessed using validation plots and calibration statistics (calibration-in-the-large) [3]. In the validation plots predicted vs. observed outcomes are plotted to depict the deviation of their agreement from the optimal situation. Calibration-in-the-large was assessed by fitting a logistic regression model with the model predictions as an offset variable and by calculating the intercept and calibration slope. The intercept was used to assess whether the model predictions are systematically too low or too high. In case of perfect calibration-in-the-large, i.e. the percentage predicted outcome is equal to the percentage observed, the intercept is 0. If outcomes are better than predicted, the intercept is negative. The calibration slope, which in an ideal case equals 1, was used to quantify the average strength of the predictors, compared to the development dataset.

## Results

### Comparison of datasets

The INRO-PH dataset was compared side by side with datasets used to develop the IMPACT, CRASH and Nijmegen models. Table 2 presents the description of predictor variables used in all models and their equivalent in the INRO-PH dataset. Compared to the IMPACT dataset the patients in the INRO-PH dataset were significantly older (median of 50 vs. 30 years) with higher proportions of EDH and tSAH, higher proportion of reactive pupils (71% vs 63%) and similar distribution of GCS motor scores (scores 1-3 in 44% vs. in 41% in the IMPACT dataset). Both mortality and the proportion of unfavourable outcome were lower in the INRO-PH dataset.

**Table 2:** **Characteristics of the datasets used to create the prognostic models and the INRO-PH validation dataset**

**Model**	**Measure or Category**	**INRO-PH**	**IMPACT**	**CRASH**	**Nijmegen: RUBICS**		
**Variable**					**Moderate**	**Severe**	**Total**
**N**		778	8,509	10,008	126	574	700
**Age (median, IQR)**	Years	50 (28-69)	30 (21-45)	-	-	-	
**Age (mean, SD)**	Years	49 (23)	-	37 (17)	48 (22)	43 (20)	
**GCS at ED (median, IQR)**	Points	3 (3-8)	-	-	11	3	
**GCS at randomization (N, %)**	Mild (13-14)	-	-	30%	-	-	
	Moderate (9-12)	-	-	30%	-	-	
	Severe (3-8)	-	-	40%	-	-	
**Motor GCS on admission (N, %)**	None (1)	244 (31%)	1,395 (16%)	-	-	-	
	Extension (2)	31 (4%)	1,042 (12%)	-	-	-	
	Abnormal flexion (3)	72 (9%)	1,085 (13%)	-	-	-	
	Normal flexion (4)	133 (17%)	1,940 (23%)	-	-	-	
	Localizes/obeys (5/6)	218 (28%)	2,591 (30%)	-	-	-	
	Untestable/missing	80 (10%)	456 (5%)	-	-	-	
**Pupillary reactivity (N, %)**	Both pupils reactive	371 (71%)	4,486 (63%)	83%	117 (93%)	374 (65%)	491 (70%)
	One pupil reactive	31 (6%)	886 (12%)	7%	6 (5%)	74 (13%)	80 (11%)
	No pupil reactive	123 (23%)	1,754 (25%)	8%	3 (2%)	126 (22%)	129 (18%)
	Unable to assess	8 (1%)	-	3%	-	-	
**Major extracranial injury (N, %)**	Present	389 (50%)	-	23%	-	-	
**Hypoxia (N, %)**	Yes or suspected	117 (15%)	1,116 (20%)	-	12 (10%)	165 (29%)	177 (25%)
**Hypotension (N, %)**	Yes or suspected	103 (13%)	1,171 (18%)	-	7 (6%)	133 (23%)	140 (20%)
**CT classification (N, %)**	Diffuse Injury (I)	84 (11%)	360 (7%)	-	48 (40%)	104 (19%)	152 (22%)
	Diffuse Injury (II)	327 (44%)	1,838 (35%)	-	38 (31%)	156 (29%)	194 (28%)
	Diffuse Injury (III)	55 (7%)	863 (17%)	-	5 (4%)	88 (16%)	93 (13%)
	Diffuse Injury (IV)	3 (1%)	187 (4%)	-	0	17 (3%)	17 (2%)
	Mass lesion evacuated (V) or non-evacuated (VI)	270 (37%)	1,944 (38%)	-	30 (25%)	172 (32%)	202 (29%)
**tSAH**	Yes	437 (56%)	3,313 (45%)	32%	-	-	
**EDH**	Yes	128 (16%)	999 (13%)	-	-	-	
**Six month outcome (N, %)**	Dead	212 (27%)	2,396 (28%)	2,146 (32%)^a^	29 (23%)	222 (39%)	251 (36%)
	Unfavourable	265 (36%)	4,082 (48%)	3,139 (47%)^a^	39 (31%)	289 (50%)	328 (47%)

^a^For subset of patients in the CRASH dataset with GCS < =12; GCS = Glasgow Coma Scale; tSAH = Traumatic Subarachnoid Haemorrhage; EDH = Epidural hematoma; Data for IMPACT [13], CRASH [6] and RUBICS [11] datasets extracted from original publications-format of data presentation differs in some cases.

About one third of patients in the CRASH dataset had a mild TBI whereas all patients had moderate or severe TBI in the INRO-PH dataset. Thus, in general the severity was lower in the CRASH patients. The mean age was higher in the INRO-PH dataset (mean 49 vs. 37 years). The outcomes in the subset of CRASH patients with moderate or severe TBI were worse than in the INRO-PH patients.

The data for the RUBICS dataset (used to develop the Nijmegen models) was reported separately for moderate and severe TBI and where possible it was summarized. In general, patients in the RUBICS dataset were slightly younger and had less severe injuries (higher median of total GCS, lower proportion of mass lesions) but had worse outcomes compared to the INRO-PH dataset.

### Predictor effects

A detailed comparison of predictor effects (using ORs) reported with the original models and in the models refitted using the INRO-PH dataset is presented in Table 3. Age as a predictor was included in all models and its effects were similar in all comparisons. The ORs of the effect of the summary GCS in the models and refits were identical both in case of CRASH and Nijmegen models. The effects if GCS motor scores 1-4 in case of the IMPACT core and extended models were stronger in models for prediction of unfavourable outcome; the differences were smaller in case of mortality prediction. A generally strong effect of none reactive pupils observed in the refitted models was similar to values in the Nijmegen models (15.3 vs. 13.2 for mortality and 9.3 vs. 10.9 for unfavourable outcome) but differed from the values in the IMPACT and CRASH models where this effect was smaller. Compared to the IMPACT extended models, stronger effects of hypoxia and Marshall CT classification were observed in the INRO-PH dataset.

**Table 3:** **Predictors effects on outcomes in the prognostic models and in the refitted models on INRO-PH validation dataset (Odds Ratios)**

**Variable**	**Measure or Category**	**IMPACT Core (mortality)**		**IMPACT Core (unfavourable)**		**IMPACT Extended (mortality)**		**IMPACT Extended (unfavourable)**		**Crash basic (mortality)**		**Crash basic (unfavourable)**		**Nijmegen DCM (mortality)**		**Nijmegen DCM (unfavourable)**	
		**M**	**R**	**M**	**R**	**M**	**R**	**M**	**R**	**M**	**R**	**M**	**R**	**M**	**R**	**M**	**R**
**Age, years > 16**	OR per year	1.04	1.05	1.04	1.04	1.03	1.04	1.03	1.04	-	-	-	-	1.06	1.06	1.05	1.04
**Age, years >40**	OR per year	-	-	-	-	-	-	-	-	1.07	1.07	1.08	1.07	-	-	-	-
**GCS**	OR per point increase	-	-	-	-	-	-	-	-	0.8	0.8	0.8	0.8	0.9	0.9	0.9	0.9
**Motor GCS**	None (1)	4.3	3.9	4.0	3.4	3.3	2.1	3.4	2.5	-	-	-	-	-	-	-	-
	Extension (2)	4.0	4.7	8.0	2.7	3.3	2.7	6.4	2.3	-	-	-	-	-	-	-	-
	Abnormal flexion (3)	2.2	2.2	3.5	2.1	2.1	1.7	3.3	1.5	-	-	-	-	-	-	-	-
	Normal flexion (4)	1.5	1.7	1.9	1.4	1.4	1.6	1.8	1.2	-	-	-	-	-	-	-	-
	Localizes/obeys (5/6)	Ref	Ref	Ref	Ref	Ref	Ref	Ref	Ref	-	-	-	-	-	-	-	-
	Untestable / missing (9)	1.7	3.9	2.4	3.3	1.5	1.9	2.3	1.6	-	-	-	-	-	-	-	-
**Pupillary reactivity**	Both pupils reactive	Ref	Ref	Ref	Ref	Ref	Ref	Ref	Ref	Ref	Ref	Ref	Ref	Ref	Ref	Ref	Ref
	One pupil reactive	1.7	1.3	1.8	1.5	1.4	1.6	1.6	1.6	3.1	1.2	2.6	1.1	1.9	1.14	1.8	1.2
	No pupil reactive	3.4	15.5	3.4	8.9	2.6	10.2	2.7	5.2	5.3	12.2	3.3	7.7	13.2	15.3	10.9	9.3
**Major extra-cranial injury**	Yes	-		-	-	-	-	-	-	1.4	1.1	1.6	1.1	-	-	-	-
**Hypoxia**	Yes or suspected	-		-	-	1.3	2.1	1.4	2.2	-	-	-	-	-	-	-	-
**Hypotension**	Yes or suspected	-		-	-	2.0	2.4	1.9	2.1	-	-	-	-	2.2	1.5	3.1	2.0
**CT classification**	Diffuse injury (I)	-		-	-	0.7	0.6	1.7	0.5	-	-	-	-	-	-	-	-
	Diffuse injury (II)	-		-	-	Ref	Ref	Ref	Ref	-	-	-	-	-	-	-	-
	Diffuse injury (III/IV)	-		-	-	2.2	5.3	1.7	2.7	-	-	-	-	-	-	-	-
	Mass lesion evacuated or non-evacuated (V/VI)	-		-	-	1.9	6.4	1.6	6.5	-	-	-	-	-	-	-	-
**tSAH**	Yes	-		-	-	1.8	1.4	1.8	1.3	-	-	-	-	-	-	-	-
**EDH**	Yes	-		-	-	0.7	0.7	0.6	0.6	-	-	-	-	-	-	-	-

M = model; R = refitted; GCS = Glasgow Coma Scale; tSAH = Traumatic Subarachnoid Haemorrhage; EDH = Epidural Hematoma; Ref = reference category; OR = odds ratio.

### Model performance

All models showed a good ability to discriminate between survival and death and between favourable and unfavourable outcome, as indicated by values of AUC_VAL_ (Table 4). In all validations, the AUC was 0.8 or higher. In all cases higher values were achieved for prediction of mortality than for unfavourable outcome. Although the Nijmegen models showed the best discriminative ability for both outcomes, the difference in AUC_VAL_ compared to other models were small (0.01-0.02 points in case of unfavourable outcome and 0.01-0.04 in case of mortality) and non-significant.

**Table 4:** **Performance measures of the prognostic models on the INRO-PH validation dataset**

**Performance measures (prediction of six-month unfavourable outcome)**	**AUC**_**VAL**_ **(CI 95%)**	**AUC**_**REFIT**_	**Slope**	**Intercept**	P- value*
**IMPACT Core Model**	0.8 (0.76-0.84)	0.82	1.073	-0.354	0.93
**IMPACT Extended Model**	0.81 (0.77-0.86)	0.87	1.061	-0.506	0.65
**CRASH Basic Model**	0.8 (0.75-0.84)	0.82	0.871	-0.505	Reference
**Nijmegen Demographic and Clinical Model**	0.82 (0.77-0.87)	0.83	0.865	-0.738	0.47
**Performance measures (prediction of six-month mortality)**	**AUC**_**VAL**_	**AUC**_**REFIT**_	**Slope**	**Intercept**	P- value*
**IMPACT Core Model**	0.84 (0.8-0.87)	0.86	1.381	-0.211	0.55
**IMPACT Extended Model**	0.85 (0.81-0.89)	0.9	1.323	-0.243	0.32
**CRASH Basic Model**	0.82 (0.77-0.87)	0.85	0.866	0.207	Reference
**Nijmegen Demographic and Clinical Model**	0.86 (0.82-0.91)	0.87	0.984	-0.289	0.19

AUC _VAL_ = Area under the curve of external validation; AUC _REFIT_ = Area under the curve of the refitted models; CI = Confidence Interval.

*P value refers to difference between AUC _VAL_: The model with the lowest AUC_VAL_ was taken as a reference and tested with all other AUC_VAL._

The difference between AUC_VAL_ and AUC_REFIT_ reflected the extent to which the effects of predictors in the original models were suboptimal. The AUC_REFIT_ shows the maximum discrimination achievable using the INRO-PH dataset, given the specification of predictors in each of the original models. For all models, the performance was better after refitting, with AUCs of 0.82 or higher.

**Figure 1:**
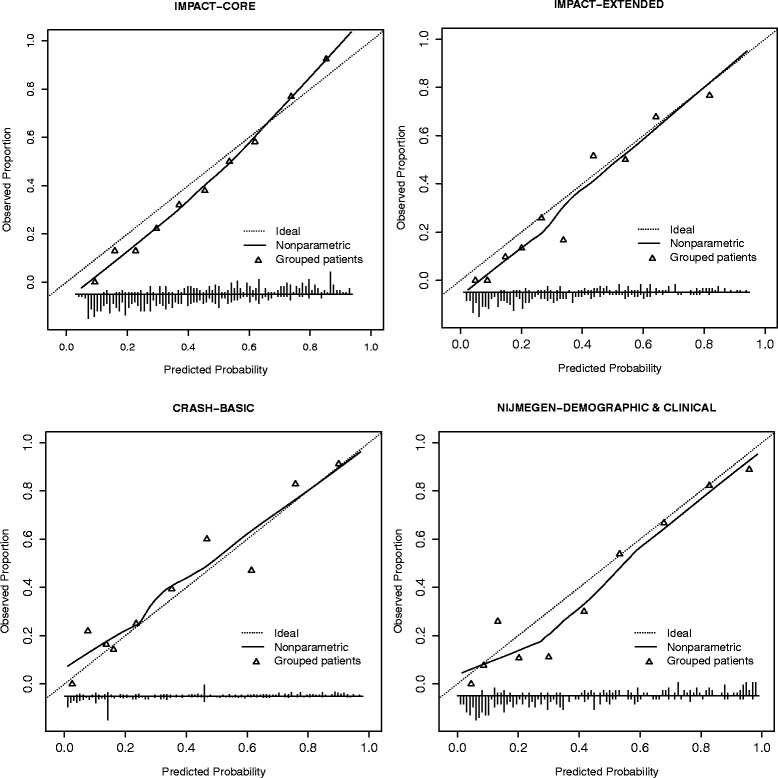
**Calibration plots for prognostic models predicting 6 month mortality.** In the figures predicted probabilities of mortality or unfavourable outcome are plotted against actual observed proportions and this relationship is shown as a curve. The dotted diagonal line shows the optimal shape of the curve where the predicted and observed values match. The triangles show observed proportions by decile of predicted probability.

**Figure 2:**
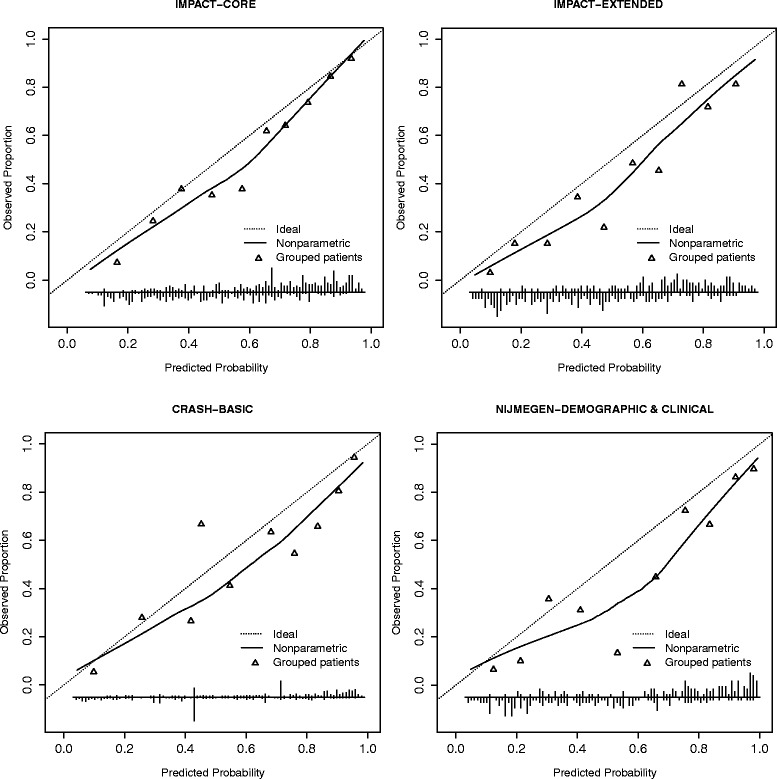
**Calibration plots for prognostic models predicting 6 month unfavourable outcome.**

The calibration plots (Figures 1 and 2) indicate systematic under prediction in all cases and a better calibration of models for prediction of mortality compared to the models for prediction of unfavourable outcome. Except for the CRASH mortality model (intercept 0.207), all observed frequencies of unfavourable outcome and death were better than predicted (intercept <0). Calibration slopes were close to 1 in all cases (0.865-1.073 in case of unfavourable outcome and 0.866-1.381 in case of models for prediction of mortality), indicating good calibration. The IMPACT extended model had the slope closest to 1 (1.061) for prediction of unfavourable outcome and for mortality prediction the slope of the Nijmegen model was closest to 1 (0.984).

## Discussion

We conducted a study to externally validate three sets of prognostic models for prediction of outcome in patients with moderate or severe TBI using a recently (2009-2012) collected observational dataset of 778 TBI patients from Austria. We focused on recently developed models that fulfilled high methodological standards for their development. We confirmed the external validity of the IMPACT and CRASH models in predicting outcome of patients with moderate or severe TBI in the INRO-PH database of Austrian TBI patients. In addition, our results showed good performance of the more recently developed Nijmegen models. The overall performance of all models in cross-comparison was similar as indicated by calibration and discrimination measures.

All models were externally validated before as part of their development. The IMPACT models were externally validated on a set of 6,681 patients from the CRASH trial; CRASH models have been externally validated using the IMPACT dataset; and the Prospective Observational Cohort Neurotrauma [16] dataset was used to validate the Nijmegen models.

The IMPACT core and CRASH basic models were additionally externally validated side by side using 5 different datasets (3 RCTs and 2 observational studies) [10]. Furthermore, both the IMPACT core and extended models were validated using the Prospective Observational Cohort Neurotrauma [16] dataset of 415 cases [7] and using a dataset of 587 patients with prospective consecutive data collection [8]. All three studies concluded that the models performed well on the validation datasets. Another study validated the IMPACT models on a set of 342 patients from a retrospective single centre study and also confirmed their good performance [9].

Our findings contribute further to the evidence base on the performance of prognostic models for TBI patients. Specifically, we observed a better than expected outcome in recent patients. We note however that caution must be used when interpreting our findings because factors beyond the model and its development must be considered.

First, the discrimination of the model on external datasets is highly sensitive to case-mix of patients - substantially higher AUCs were reported when using observational dataset compared to RCTs due to generally less restricted patient enrolment criteria [10].

Second, the calibration of the models is influenced by the effect of predictors and by the distribution of outcomes. A previous study reported that miscalibration could occur due to differences in outcome of patients on the GOS scale. This was found to be U-shaped in the IMPACT and CRASH development datasets (e.g. relatively many patients died or had favourable outcome, and relatively few patients were vegetative or had severe disability) and differed from the distribution in their validation datasets [10]. In our patients, the distribution of outcomes was similarly U-shaped as in the IMPACT and CRASH studies. We can therefore assume that in our study the miscalibration was at least to some extent influenced by the different predictor effects (see Table 3 for details).

Third, additional factors that influence the overall performance of the models include system factors such as trauma care organization or treatment policies. We can speculate that the advances in treatment could be the cause of better than predicted outcomes, although there is no clear evidence to support this presumption [17,18]. A number of specific factors could be relevant here: A) patients in the INRO-PH dataset were treated by well-trained emergency physicians in the field, and the mean interval between arrival of Emergency Medical Service teams and arrival at hospital was only 51 minutes; B) the short intervals between hospital admission and first CT scan (median of 25 minutes), and between CT scan and surgery (median between ER admission and surgery was 93 minutes); and C) a high proportion of patients had advanced management of possible coagulation disorders [19].

Influences of case-mix of patients, predictor effects, outcome distribution and improvements of care could be tackled by adjusting the models [20].

Nevertheless, our study and previous evidence [7-10,13] suggests that the IMPACT, CRASH and Nijmegen models are valid and useful tools that can be effectively used for predicting the risk of adverse outcome in patients with TBI. Such prediction is relevant in clinical settings as well as research where quantification of expected outcomes of patients can be helpful in assessing quality of care and evaluating the effectiveness of certain procedures (e.g. by comparing expected vs. observed outcome). Furthermore, they could be used to inform relatives of patients, to make treatment decisions or to decide on allocation of resources [7].

From the practical point of view, two aspects could be considered when deciding on the model to be used. First, the complexity of the model. In general, more complex models (e.g. those including clinical variables such as CT scan findings) have better discriminative ability [7,10]. However, in our study the performance of more complex models (IMPACT extended models) was not much better than that of simpler models. In fact, the IMPACT core model, the simplest model validated in this study which uses only three predictors, performed similarly to all other models. Another dimension to this aspect of a model is that the more predictors are needed the higher chance that some will be missing - making predictions impossible. Thus, models with less predictors could be preferred over more complex models as we can assume that the information for the prediction will be easier to obtain and such models perform similarly to more complex models. Second, the actual usability of the model is tested by external validation studies using various populations, such as in our study. Therefore, more external validations could mean higher confidence in the performance of the model. In our study the Nijmegen models performed best (although the differences compared to other models were small). However, in the time of writing this paper these models were not externally validated as widely as the IMPACT or CRASH models and thus recommending their preference over other models should be cautious.

The fact that a number of different prognostic models for prediction of outcome in patients with TBI are available with different sets of predictors and outcomes used provides clinicians and researchers with a range of choices. The decision on which model to use in specific settings and situations could be made according to the above mentioned factors and will be in most situations driven by availability of predictor values and/or the desired outcome to be predicted. In order to maintain and extend the range of available models it is of cardinal importance that the models will be continuously validated in external settings and updated using more recent data.

We acknowledge that this study has some limitations. *S*ome measures and variables may not exactly match with the development data. For example, some measures differ as to the timing of their assessment (e.g. GCS at randomization in the CRASH dataset is compared to GCS at trauma room in the INRO-PH dataset). Furthermore, there might be other differences such as methodology of assessment of six month outcome, which tends to differ between studies, related to outcome distribution and predictor effects [10].

In the CRASH model a relatively loose definition of MEI is used: “an injury requiring hospital admission within its own right” [21]. Such a broad definition may include various types of injuries in various settings. In our study we defined MEI as any injury with an AIS > 2 and thus the actual injuries considered as MEI in CRASH and in our study may differ.

The findings of this study should not be generalized without cautious considerations. Prognostic models should be seen as tools to predict outcomes in clusters of patients with certain characteristics and may thus aid clinical, research and policy work. The findings of this study inherit these characteristics and they should be interpreted and used in such context.

In conclusion, all the prognostic models we validated in this study possess good discriminative ability for prediction of six month outcome in patients with moderate or severe TBI but outcomes were systemically better than predicted. After adjustment for this under prediction in locally adapted models, these may well be used for recent TBI patients.
